# Neutrophil CD64 index for diagnosing and tracking treatment response across the mycobacterial infection spectrum: a retrospective two-cohort study

**DOI:** 10.3389/fmicb.2026.1980277

**Published:** 2026-09-25

**Authors:** Yuxi Chen, Xu Ma, Lei Wang, Yankun Li, Kun Tong, Jinghui Yang, Fangyou Yu, Xiaocui Wu

**Affiliations:** 1 Department of Clinical Laboratory Medicine, Shanghai Pulmonary Hospital, School of Medicine, Tongji University, Shanghai, China; 2 Department of Tuberculosis, Shanghai Pulmonary Hospital, School of Medicine, Tongji University, Shanghai, China; 3 Department of Pathology, Shanghai Pulmonary Hospital, School of Medicine, Tongji University, Shanghai, China; 4 Shanghai Key Laboratory of Tuberculosis, Shanghai Pulmonary Hospital, School of Medicine, Tongji University, Shanghai, China

**Keywords:** biomarker, differential diagnosis, mycobacterial infection, neutrophil CD64, non-tuberculous mycobacteria (NTM), tuberculosis

## Abstract

**Background:**

The neutrophil CD64 (nCD64) index has emerged as a promising biomarker for infection detection, but its evaluation within the mycobacterial infection spectrum remains limited. Here, we evaluated the diagnostic and treatment response performance of the nCD64 index in the active tuberculosis (ATB)—including pulmonary tuberculosis (PTB) and extrapulmonary tuberculosis (ePTB), and non-tuberculous mycobacterial (NTM) disease, and determined whether it adds value beyond standard inflammatory indicators.

**Methods:**

We measured the nCD64 index in a cross-sectional cohort comprising 248 participants across five groups: ATB, latent tuberculosis infection (LTBI), NTM, bacterial pulmonary infection (PI) and healthy donors (HD). Patients with ATB and NTM had not received anti-mycobacterial treatment at the time of enrollment. Diagnostic performance was assessed by receiver operating characteristic (ROC) curve analysis, and incremental value beyond white blood cell (WBC) count and C-reactive protein (CRP) was evaluated by comparing an area under the curve (AUC) with and without nCD64 index. In a separate longitudinal cohort of 244 participants, nCD64 index was measured at baseline and months 1–3 of anti-mycobacterial therapy, and longitudinal trajectories were compared between treatment-naive and retreatment patients using a linear mixed-effects (LME) model.

**Results:**

nCD64 index was markedly elevated in ATB compared to all other groups (all *p* < 0.001). It discriminated ATB from LTBI with an AUC of 0.739 and PTB from NTM with an AUC of 0.744. Addition of nCD64 index to WBC count significantly improved AUC in four diagnostic comparisons with gains of 0.12 to 0.34, most markedly for PTB versus PI. nCD64 index was significantly higher in PTB than NTM (*p* < 0.001) with limited stratification by clinical features. In the longitudinal cohort, nCD64 index declined progressively from month 1 in both PTB and NTM patients and remained lower through month 3. Treatment-naive patients exhibited significantly steeper decline trajectories than retreatment patients (PTB *p* < 0.001, NTM *p* = 0.004).

**Conclusion:**

The nCD64 index discriminates ATB from LTBI and NTM, adds diagnostic value beyond routine inflammatory markers, and tracks serial changes during treatment in both ATB and NTM. Treatment-naive patients showed more pronounced decline trajectories, supporting particular utility of the nCD64 index for tracking initial treatment response. It warrants further evaluation as a complementary biomarker in the clinical management of mycobacterial infections.

## Introduction

Tuberculosis (TB), caused by *Mycobacterium tuberculosis*, remains a major global health threat, with an estimated 10.8 million incident cases and 1.25 million deaths in 2024 (World Health Organization, 2025). The clinical management of tuberculosis depends on the ability to accurately distinguish between active tuberculosis (ATB)—including pulmonary tuberculosis (PTB) and extrapulmonary tuberculosis (ePTB), in which lesions involve the pleura, lymph nodes, meninges, or bones—and latent tuberculosis infection (LTBI) and non-tuberculous mycobacterial (NTM) disease. The prevalence of NTM is on the rise globally and shows significant overlap with PTB in terms of clinical and radiological presentations (Ratnatunga et al., 2020). Existing diagnostic tools have practical limitations: culture-based methods are slow and insensitive in paucibacillary disease; interferon-*γ* release assays (IGRA) detect *M. tuberculosis* sensitization but cannot distinguish ATB from LTBI and cross-react with several NTM species; and conventional inflammatory markers (CRP, WBC count, ESR) are non-specific (Suárez et al., 2019). Recent studies on blood-based host-response biomarkers have demonstrated considerable potential for PTB diagnosis. An optimized 5-gene pair classifier showed high diagnostic accuracy with an AUC of 0.947 and robust diagnostic performance in HIV-coinfected individuals (Wu et al., 2026a). Another study indicated that a large scale non-invasive, blood transcriptome-based diagnostic model of 2,792 samples demonstrated consistently strong performance across diverse geographic and clinical settings, supporting the potential of host-response transcriptomic signatures for TB diagnosis (Wu et al., 2026b). Together, these studies underscore the growing value of host-response biomarkers and provide a strong rationale for exploring measurable biomarkers.

CD64, an immunoglobulin (Ig) G Fc receptor (Fcγ RI) (Holtrop et al., 2025), is constitutively expressed on monocytes and macrophages, and is present at negligible levels on resting neutrophils under normal conditions. During active infection, proinflammatory cytokines—including interferon-*γ* (IFN-*γ*) and granulocyte colony-stimulating factor (G-CSF)—rapidly upregulate CD64 on the neutrophil surface within 4–6 h of stimulation (Bourgoin et al., 2020). The neutrophil CD64 (nCD64) index normalizes neutrophil CD64 expression against monocyte and lymphocyte reference channels, reducing inter-individual variability and enabling standardized cross-platform measurement (Icardi et al., 2009). The nCD64 index has demonstrated clinical utility in sepsis and bacterial infections (Cui et al., 2021; Liu et al., 2022; Huang et al., 2024), but its evaluation in mycobacterial infection remains limited. Prior studies in TB patients reported elevated nCD64 index in PTB and ePTB with decline following anti-mycobacterial therapy (Liu et al., 2020), and discriminatory capacity between ATB and LTBI (Corrêa et al., 2019). However, almost none included NTM disease as a comparator, and none assessed nCD64 index as a longitudinal treatment-response indicator in NTM.

In the present study, we evaluated the nCD64 index in a treatment-naive cross-sectional cohort comprising five groups: ATB, LTBI, NTM, bacterial pulmonary infection (PI), and healthy donors (HD). In a separate longitudinal cohort, we tracked nCD64 index over 3 months of therapy in patients with PTB, ePTB, and NTM. Our goals were to define the diagnostic positioning of nCD64 index across the mycobacterial infection spectrum, to assess whether it adds diagnostic value beyond conventional inflammatory markers, and to determine its utility as a treatment response indicator.

## Materials and methods

### Study design and participants

This retrospective observational study was conducted at Shanghai Pulmonary Hospital of Tongji University, a tertiary-care referral center specializing in pulmonary diseases and a national designated tuberculosis hospital. The study was approved by the Institutional Ethics Committee of SPH (No: K25-671Z). Informed consent requirements were waived due to the retrospective study design.

Patients included in the study were inpatients from June 2025 to March 2026. The cross-sectional cohort comprised five diagnostic groups. Inclusion criteria were age ≥17 years and HIV-negative status confirmed prior to inclusion. The exclusion criteria were as follows: (1) unknown final diagnosis; (2) unavailability of nCD64 index; (3) combination of fungal infections and severe pneumonia; (4) concurrent tumor/cancer immunotherapy. All ATB and NTM patients were treatment-naive at the time of initial clinical evaluation. An independent longitudinal cohort of patients was followed at baseline (M0) and months 1, 2, and 3 (M1–M3) of standard antimicrobial therapy over the same study period. Both treatment-naive and retreatment patients were included to reflect clinical practice and to allow subgroup analysis of treatment response. Retreatment patients were enrolled if they had prior exposure to anti-mycobacterial therapy and were re-initiating treatment at study entry due to relapse, re-infection, or premature self-discontinuation owing to adverse drug reactions with subsequent symptom progression, as determined by the treating clinician based on clinical assessment (Fentie et al., 2020). Only patients with a valid M0 baseline measurement and at least one follow-up visit were included in the longitudinal cohort. The exclusion criteria were as follows: (1) missing follow-up data of nCD64 index; (2) combination of severe infection; (3) concurrent tumor/cancer immunotherapy. This cohort was entirely independent of the cross-sectional cohort, with no patient overlap. The flowchart is shown in Figure 1.

**Figure 1:**
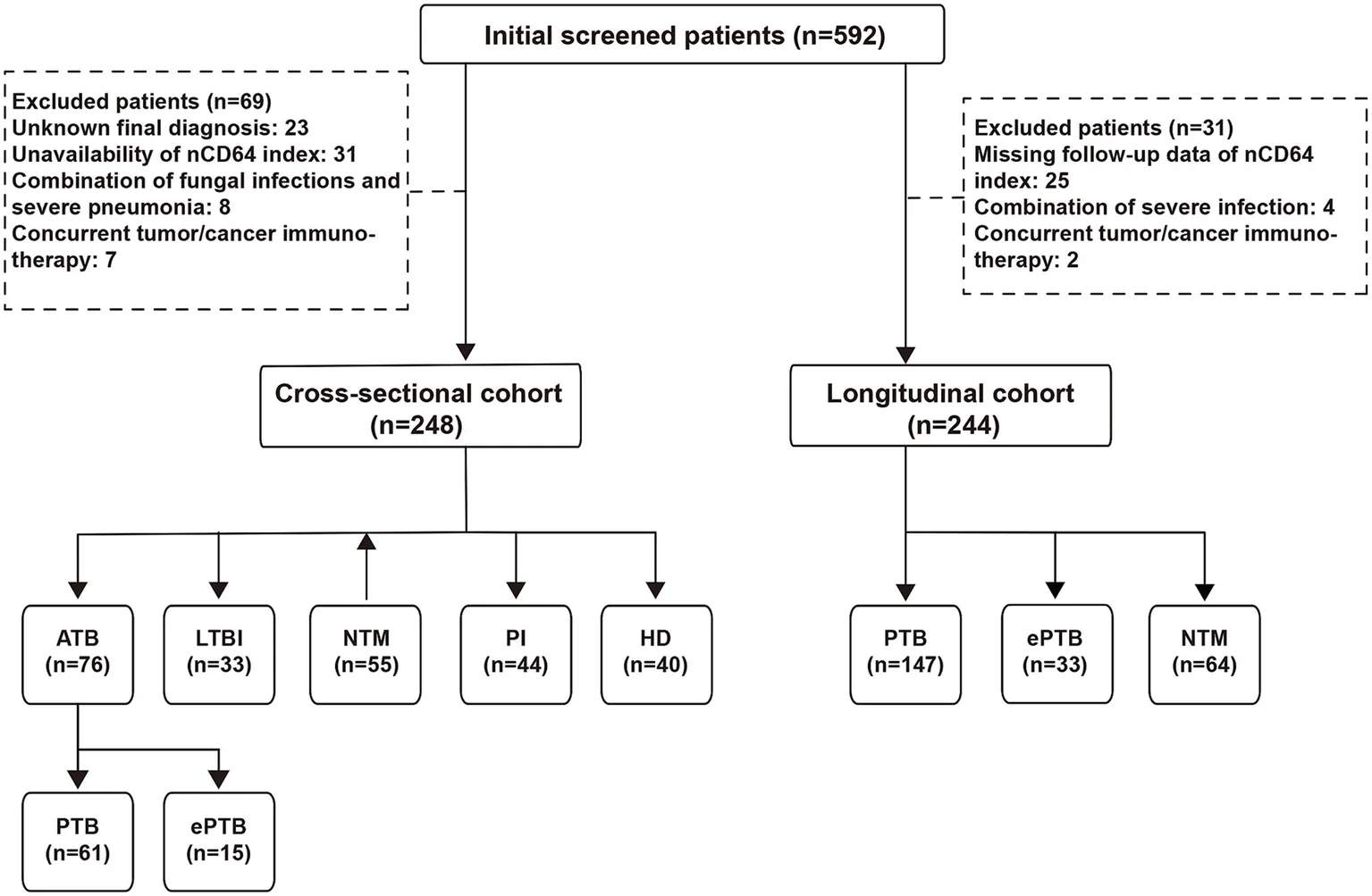
Flowchart of the included patients.

### Diagnostic criteria

All microbiological testing (smear, culture, IGRA, Xpert MTB/RIF) was performed at the clinical laboratory of SPH, an ISO 15189 accredited laboratory specializing in MTB detection. Smears were examined using fluorescence staining, and liquid culture was performed using the BACTEC MGIT 960 system (Becton Dickinson, Cockeysville, MD, USA).

ATB was diagnosed by microbiological confirmation (positive sputum or lavage fluid culture, smear, Xpert MTB/RIF (Cepheid, Sunnyvale, CA, USA), or other nucleic acid amplification test) in conjunction with compatible radiographic abnormalities and clinical diagnosis of tuberculosis (Acharya et al., 2020). In culture-negative cases, diagnosis was based on clinical and radiological criteria with a positive response to empirical anti-mycobacterial therapy. ePTB comprised localized extrapulmonary TB manifestations, including lymph node, urological, osseous, and other non-pulmonary sites, cases with disseminated TB, tuberculous pleuritis, or tuberculous meningitis were excluded from this subgroup. LTBI was defined as a positive IGRA result in asymptomatic individuals with a normal chest radiograph and no clinical evidence of active TB; participants were excluded if they developed tuberculosis disease within 6 months of enrollment, to avoid misclassifying early asymptomatic disease as LTBI (Zak et al., 2016; Wu et al., 2023). NTM was diagnosed based on the 2020 ATS/ERS/ESCMID/IDSA clinical practice guidelines (Daley et al., 2020), requiring at least two positive sputum cultures or one positive bronchoscopic specimen. PI was defined as community-acquired bacterial pneumonia confirmed by clinical, laboratory, and chest imaging criteria. HD was recruited from a routine health examination program conducted at our institution, with no history of tuberculosis, no known TB contact, no recent respiratory symptoms, and normal chest imaging. Patients in the longitudinal cohort were enrolled on the basis of confirmed mycobacterial disease and initiation of standard anti-mycobacterial treatment. Treatment responsiveness in the longitudinal cohort was assessed by the treating clinician at each follow-up visit based on symptomatic improvement and radiological changes on chest imaging.

### nCD64 index measurement

Peripheral EDTA-K2-anticoagulated blood samples were collected within 24 h of admission and processed at room temperature within 24 h of collection. Whole blood (50 μL) was incubated with CD45-PerCP, CD14-FITC, and CD64-PE antibodies (BD Biosciences, San Jose, CA, USA) for 15 min, followed by red blood cell lysis. CD64 expression was measured by flow cytometry (FACSCanto plus, BD Biosciences), and median fluorescence intensity (MFI) values for neutrophils, monocytes, and lymphocytes were extracted using FlowJo™ Software 10. The nCD64 index was calculated as: nCD64 index = (neutrophil CD64 MFI/lymphocyte CD64 MFI)/(monocyte CD64 MFI/neutrophil CD64 MFI). Sample stability has been validated at room temperature for up to 24 h (Liu et al., 2020).

### Co-measured inflammatory indicators and clinical severity features

Concurrent measurements of C-reactive protein (CRP), white blood cell (WBC) count, erythrocyte sedimentation rate (ESR), heparin-binding protein (HBP), and soluble growth stimulation expressed gene 2 (ST2) were obtained from routine clinical samples at the same time point as nCD64 index measurement. Data on pulmonary cavitation, pleural effusion and hemoptysis in patients with PTB and NTM were retrieved from baseline chest computed tomography (CT) reports and clinical diagnosis records at admission, and were recorded as clinical severity features.

### Statistical analysis

All nCD64 index values were log10-transformed prior to analysis and are displayed on a log10 scale throughout. Group comparisons were performed using the Kruskal–Wallis test with Dunn’s correction (multiple groups) or Wilcoxon rank-sum test (two groups). Spearman rank correlation was used to assess associations between nCD64 index and co-measured inflammatory indicators. Diagnostic performance was evaluated by ROC curve analysis, 95% confidence intervals for AUC were computed by DeLong’s method. For longitudinal analyses, within-group changes from baseline (M0 vs. M1, M0 vs. M2, M0 vs. M3) were assessed by paired Wilcoxon signed-rank tests, with false discovery rate (FDR) correction applied within each group. The effect of prior treatment history on baseline nCD64 index was assessed by Mann–Whitney U test. Categorical variables were compared using *χ*^2^ test or Fisher’s exact test, as appropriate. To evaluate whether prior treatment history influenced longitudinal nCD64 index trajectories, a linear mixed-effects (LME) model was fitted for each disease group (PTB, ePTB, NTM) with nCD64 index as the dependent variable, month (continuous) and treatment history (treatment-naive vs. retreatment) as fixed effects, and patient as a random intercept to account for repeated measures. The Month × treatment history interaction term was used to test whether trajectories differed between subgroups. Models were fitted using the lme4 package (version 1.1) in R (version 4.4.0). All analyses were performed in GraphPad Prism 8 (GraphPad, Inc., CA, USA) and R (version 4.4.0). Statistical significance was set at *p* < 0.05.

## Results

### Characteristics of the participants

A total of 248 participants were enrolled in the cross-sectional cohort comprising five diagnostic groups: ATB (active tuberculosis, including PTB [pulmonary TB, *n* = 61] and ePTB [extrapulmonary TB, *n* = 15]; total *n* = 76), LTBI (latent tuberculosis infection, *n* = 33), NTM (non-tuberculous mycobacterial pulmonary disease, *n* = 55), PI (bacterial pulmonary infection, *n* = 44), and HD (healthy donors, *n* = 40). Age and sex distribution differed significantly across groups (both *p* < 0.001). IGRA positivity was highest in LTBI (100%), PTB (89.8%), and ePTB (83.3%), consistent with the expected distribution of *M. tuberculosis* infection; IGRA was not performed in the HD group. GeneXpert positivity was highest in PTB (80.4%) and markedly lower in ePTB (20.0%). nCD64 index differed significantly across all groups (both *p* < 0.001). An independent longitudinal cohort of 244 patients was enrolled comprising PTB (*n* = 147), ePTB (*n* = 33), and NTM (*n* = 64). The three groups differed significantly in age and sex distribution (*p* < 0.001). The proportion of treatment-naive patients was numerically higher in PTB (44.2%) than in ePTB (36.4%) and NTM (26.6%), though the difference did not reach statistical significance (*p* = 0.051). Baseline characteristics of both cohorts are summarized in Table 1.

**Table 1:** Baseline characteristics of participants in the cross-sectional and longitudinal cohorts.

Section A: cross-sectional cohort (*n* = 248)							
Characteristics	ATB		LTBI	NTM	PI	HD^a^	*p-*value^b^
	PTB	ePTB					
*n*	61	15	33	55	44	40	
Age, median (IQR), y	51 (36–67)	59 (46–73)	58 (40–69)	63 (54–72)	64 (53–70)	38 (33–46)	<0.001
Male, *n* (%)	47 (77.0%)	7 (46.7%)	23 (69.7%)	16 (29.1%)	24 (54.5%)	21 (52.5%)	<0.001
nCD64 index	3.27 (1.10–15.49)	1.48 (0.69–9.00)	0.77 (0.38–1.20)	0.68 (0.42–1.65)	0.74 (0.27–1.82)	0.54 (0.30–0.92)	<0.001
WBC count, ×10^9^/L	6.59 (5.19–8.04)	5.06 (4.26–7.28)	5.72 (5.22–7.15)	5.61 (4.49–6.75)	5.82 (4.75–7.38)	6.11 (5.41–7.29)	0.088
CRP, mg/L	5.80 (2.05–42.20)	3.70 (1.50–28.20)	3.10 (1.00–9.10)	2.30 (1.17–7.58)	4.00 (1.60–46.10)	—	0.050
ESR, mm/h	63.00 (28.00–91.00)	35.00 (24.00–49.00)	13.00 (9.00–36.00)	30.00 (10.00–40.00)	27.00 (13.50–52.25)	—	<0.001
HBP, ng/mL	21.61 (13.90–39.95)	21.34 (13.28–67.19)	15.92 (7.94–26.84)	16.19 (9.98–32.12)	16.97 (8.75–32.87)	—	0.155
ST2, ng/mL	25.50 (18.88–34.08)	19.10 (14.05–60.40)	21.20 (17.40–26.30)	19.65 (13.93–24.17)	19.60 (15.15–30.35)	—	0.022
IGRA^c^: positive, *n* (%)	44 (89.8%)	10 (83.3%)	33 (100.0%)	5 (12.5%)	12 (31.6%)	—	<0.001
GeneXpert^d^: positive, *n* (%)	45 (80.4%)	3 (20.0%)	—	—	—	—	<0.001

Section B: Longitudinal cohort (*n* = 244)					
Characteristics		PTB	ePTB	NTM	*p-*value
*n*		147	33	64	
Age, median (IQR), y		57 (38–69)	47 (36–58)	64 (54–71)	<0.001
Male, *n* (%)		91 (61.9%)	18 (54.5%)	22 (34.4%)	0.002
Anti-TB treatment history^e^	Naive, *n* (%)	65 (44.2%)	12 (36.4%)	17 (26.6%)	0.051
	Retreatment, *n* (%)	82 (55.8%)	21 (63.6%)	47 (73.4%)	
nCD64 index (M0), median (IQR)		1.41 (0.39–3.72)	0.91 (0.47–2.47)	1.06 (0.45–2.64)	0.787

Continuous variables are presented as median (IQR); categorical variables are presented as *n* (%).

ATB, active tuberculosis; PTB, pulmonary tuberculosis; ePTB, extrapulmonary tuberculosis; LTBI, latent tuberculosis infection; NTM, non-tuberculous mycobacteria; PI, bacterial pulmonary infection; HD, healthy donors; IQR, interquartile range; WBC, count white blood cell count; CRP, C-reactive protein; ESR, erythrocyte sedimentation rate; HBP, heparin-binding protein; ST2, soluble growth stimulation expressed gene 2; IGRA, interferon-gamma release assay; M0 baseline (month 0).

^a^CRP, ESR, HBP, and ST2 were not measured in the HD group; ^b^*p*-values were determined with the Kruskal–Wallis test for continuous variables and the chi-square test for categorical variables; ^c^IGRA were available for 50 PTB, 13 ePTB, 33 LTBI, 40 NTM, and 40 PI participants; ^d^GeneXpert were available for 61 PTB and 15 ePTB participants; ^e^Treatment-naive patients were defined as those who had never received prior anti-TB therapy; retreatment patients were defined as those who had received prior anti-TB therapy for 1 month or longer (Fentie et al., 2020).

### Elevated nCD64 index in ATB

The distribution of nCD64 index across the five diagnostic groups is shown in Figure 2. The ATB group had significantly higher nCD64 index than all comparison groups (median 2.91; vs. LTBI 0.77, *p* < 0.0001; vs. NTM 0.68, *p* < 0.0001; vs. PI 0.74, *p* < 0.0001; vs. HD 0.54, *p* < 0.0001). Among the ATB group, the PTB subgroup showed numerically higher nCD64 index than the ePTB subgroup (median 3.27 vs. 1.48), although this difference did not reach statistical significance (*p* = 0.518; Figure 2), likely reflecting limited statistical efficacy due to the small ePTB subgroup (*n* = 15) and the localized nature of ePTB manifestations in our cohort.

**Figure 2:**
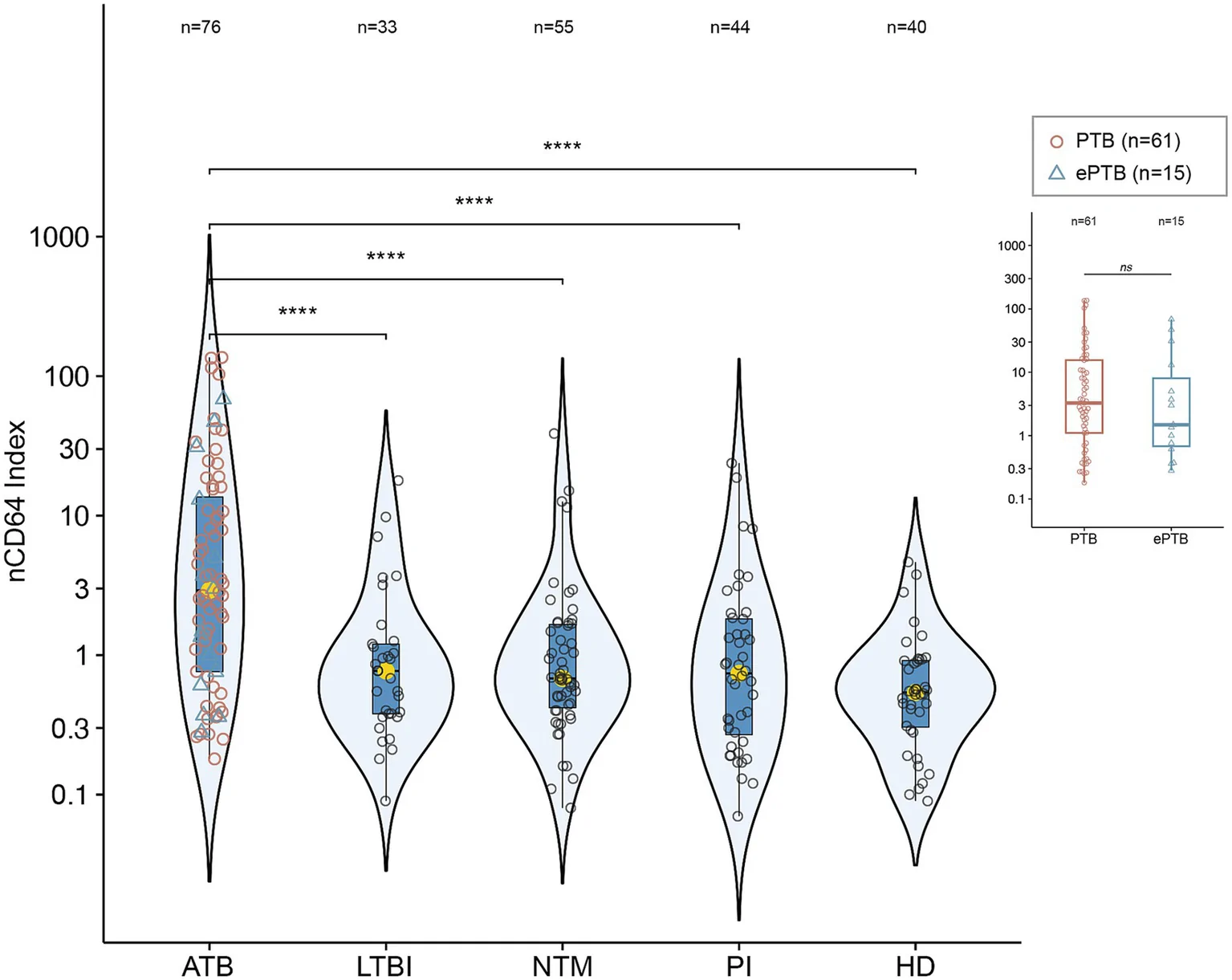
Distribution of nCD64 index across diagnostic groups in the cross-sectional cohort. Violin plots with embedded box plots display nCD64 index values (log10 scale) for ATB, LTBI, NTM, PI, and HD. Yellow diamonds indicate group medians. Significance brackets indicate pairwise comparisons between ATB and each comparison group (****p < 0.0001). Inset: comparison of nCD64 index between PTB and ePTB subgroups within the ATB group; ns, not significant.

### Diagnostic performance of nCD64 index

ROC curve analysis showed consistent discriminatory performance of the nCD64 index across all comparison groups (Figure 3A). The most clinically relevant comparison was ATB vs. LTBI, for which the nCD64 index achieved an AUC of 0.739 (95% CI: 0.643–0.834). PTB was distinguished from NTM with AUC = 0.744 (95% CI: 0.652–0.837; Figure 3B). Other optimal cutoff values, sensitivity, specificity, and likelihood ratios for each comparison are reported in Supplementary Table 1. ROC analysis for ePTB is shown in Supplementary Figure 1a.

**Figure 3:**
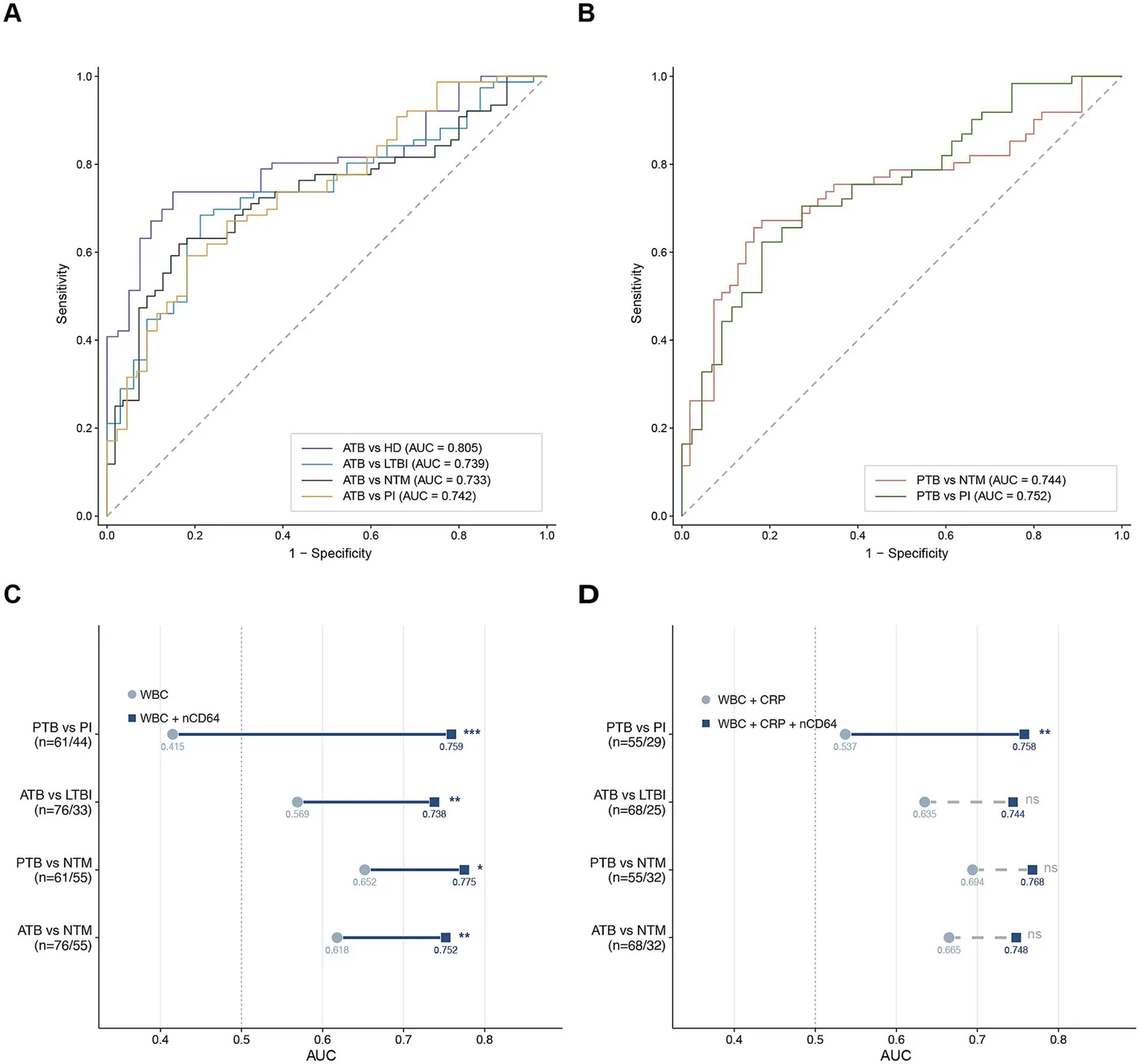
Incremental diagnostic performance of the nCD64 index beyond routine inflammatory indicators. ROC curve analysis of nCD64 index for distinguishing ATB from comparison groups **(A)** and PTB from NTM and PI groups **(B)**. AUC, area under the curve; ROC, receiver operating characteristic. Dumbbell plots show AUC for baseline models (circles) and enhanced models with nCD64 index added (squares). **(C)** WBC as baseline (full cross-sectional cohort): PTB vs. PI; ATB vs. LTBI; ATB vs. NTM; PTB vs. NTM. **(D)** WBC + CRP as baseline (CRP available subset): PTB vs. PI; ATB vs. LTBI; ATB vs. NTM; PTB vs. NTM. *p < 0.05, **p < 0.01, ***p < 0.001; ns, not significant.

### The nCD64 index augments the diagnostic performance of routine clinical inflammatory indicators

WBC count, the most widely available inflammatory indicator, showed poor discriminatory ability across the key comparisons (AUC range: 0.415–0.652). Therefore, we evaluated whether nCD64 index contributes independent diagnostic performance beyond WBC count alone and beyond WBC count combined with CRP. Adding nCD64 index to WBC count significantly improved performance across all four comparisons (Figure 3C). The largest gain was observed for PTB vs. PI (AUC: 0.415 to 0.759, *p* < 0.001), where WBC count alone performed at near chance level, followed by ATB vs. LTBI (AUC: 0.569 to 0.738, *p* = 0.004), ATB vs. NTM (AUC: 0.618 to 0.752, *p* = 0.006), and PTB vs. NTM (AUC: 0.652 to 0.775, *p* = 0.014). This incremental contribution persisted after further adjustment for WBC + CRP as the baseline (Figure 3D). Cutoff values, sensitivity, specificity, and likelihood ratios for all comparisons are reported in Supplementary Table 2.

### Correlation between the nCD64 index and routine clinical inflammatory indicators

The incremental diagnostic value of nCD64 index beyond WBC count and CRP suggests that nCD64 index and routine inflammatory indicators capture distinct biological information. To characterize this relationship, we examined the correlations of nCD64 index with several inflammatory indicators (WBC count, CRP, ESR, HBP, ST2) at the individual level. In PTB patients, nCD64 index showed significant correlations with CRP (*r* = 0.559, *p* < 0.001), ESR (*r* = 0.591, *p* < 0.001), HBP (*r* = 0.359, *p* = 0.006), and ST2 (*r* = 0.436, *p* < 0.001), but not with WBC count (*r* = 0.069, *p* = 0.595; Figure 4A). In contrast, nCD64 index showed no significant correlation with any of the five indicators in NTM patients (all *p* > 0.10, Figure 4B), suggesting that the two groups differ not only in indicator correlations but potentially in absolute indicator levels.

**Figure 4:**
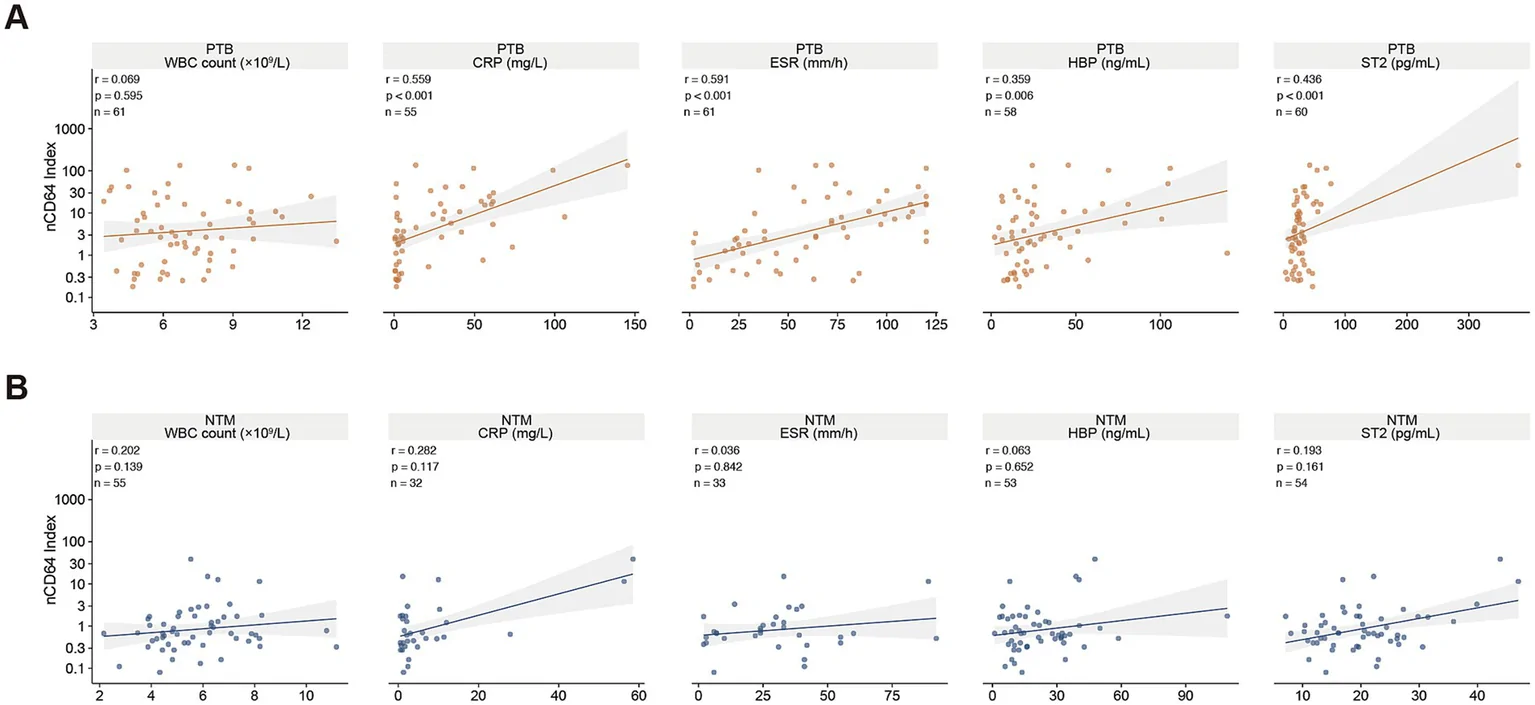
Correlations between the nCD64 index and co-measured inflammatory indicators in patients with PTB **(A)** and NTM **(B)** including WBC count, CRP, ESR, HBP and ST2. Spearman correlation analysis was used and linear curve fit (95% CI) was used to illustrate trends of data distribution. CRP, C-reactive protein; ESR, erythrocyte sedimentation rate; HBP, heparin-binding protein; ST2, soluble growth stimulation expressed gene 2. Sample sizes (*n*) vary due to missing laboratory values.

### The nCD64 index is elevated in PTB relative to NTM, with limited stratification by clinical features

Direct comparison of nCD64 index, HBP, and ST2 between PTB and NTM patients showed that nCD64 index was significantly higher in PTB than NTM (*p* < 0.001, Figure 5A), and ST2 was likewise significantly elevated in PTB relative to NTM (*p* < 0.001, Figure 5C). HBP did not differ significantly between the two groups (Figure 5B). Next, we examined whether clinical severity features within each group could further stratify marker levels. Four clinical feature comparisons (PTB: cavity and pleural effusion; NTM: hemoptysis and cavity) were evaluated across all three markers (Figure 5D). Only one of these comparisons reached statistical significance: the nCD64 index was higher in PTB with pleural effusion than in those without pleural effusion (*p* = 0.034, *r* = 0.472). No significant associations were observed for cavity in PTB or for hemoptysis and cavity in NTM (all *p* > 0.05).

**Figure 5:**
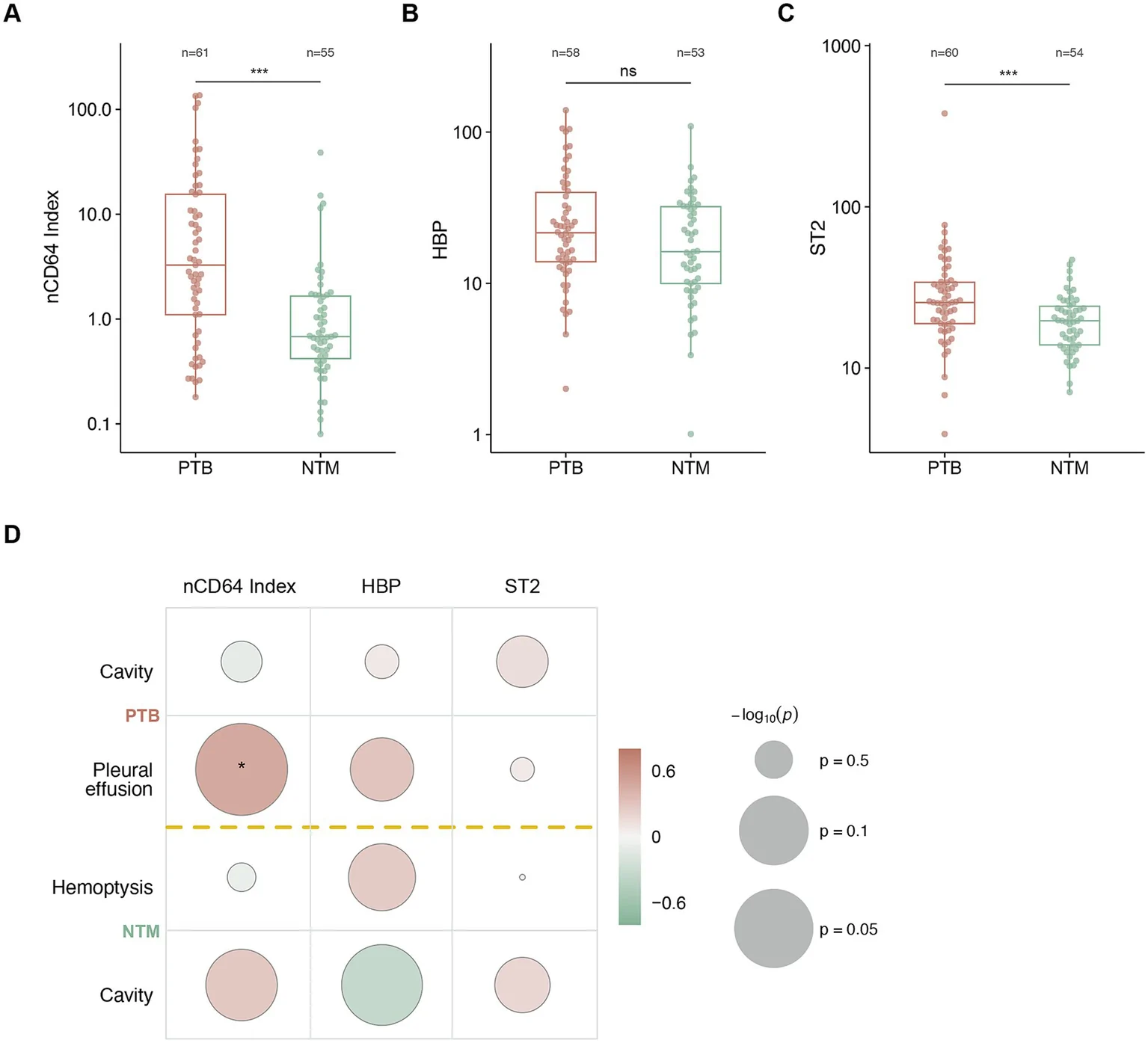
Comparison of nCD64 index, HBP, and ST2 between PTB and NTM patients, and association with clinical features. Box plots showing **(A)** nCD64 index (PTB *n* = 61, NTM *n* = 55), **(B)** HBP (PTB *n* = 58, NTM *n* = 53), and **(C)** ST2 (PTB *n* = 60, NTM *n* = 54) on a log10 scale in PTB and NTM patients. Pairwise comparisons by Wilcoxon rank-sum test, ***p < 0.001, ns: not significant. **(D)** Bubble matrix showing the association between each marker and clinical features within PTB (cavity, pleural effusion) and NTM (hemoptysis, cavity). Circle size reflects −log10(*p*); circle colour reflects rank-biserial r (red: clinical feature–positive group higher; green: clinical feature–negative group higher). Stars within circles indicate statistical significance (*p < 0.05); bubbles without stars are not significant.

### Serial changes in the nCD64 index during anti-mycobacterial treatment

To assess the performance of nCD64 index during anti-mycobacterial treatment, we retrospectively analyzed an independent cohort of PTB and NTM patients at months 1, 2, and 3 after therapy. The individual trajectories of patients in each group showed significant heterogeneity at baseline in nCD64 index, with the majority of patients exhibiting an overall downward trend (Figure 6A). Importantly, nCD64 index declined significantly as early as M1 in both groups: PTB (*p* < 0.001, *n* = 122) and NTM (*p* = 0.012, *n* = 49). In PTB, the decline remained highly significant at all three time points (M1: *p* < 0.001; M2: *p* < 0.001; M3: *p* < 0.001). In NTM, significance was likewise maintained at M2 and M3 (NTM M2: *p* = 0.002; M3: *p* = 0.037; Figure 6B). The consistent decline of nCD64 index across both infection groups supports its utility as a real-time indicator of mycobacterial clearance. In the longitudinal cohort, nCD64 index was not significantly influenced by cavitation or pleural effusion status in PTB patients (Supplementary Figure 2). In addition, retreatment PTB and ePTB patients showed significantly lower nCD64 index at M0 than treatment-naive patients (Figure 7A). In NTM, the M0 difference was non-significant (median 1.10 vs. 0.97, *p* = 0.287). Linear mixed-effects model revealed a significant Month × treatment history interaction in PTB (*p* < 0.001) and NTM (*p* = 0.004), indicating that prior treatment history influenced the pattern of nCD64 index normalization. Specifically, treatment-naive patients showed steeper decline trajectories of nCD64 index than retreatment patients in both PTB and NTM groups (Figure 7B). The same interaction was not significant in ePTB (*p* = 0.079), likely reflecting limited statistical power given the small group sizes.

**Figure 6:**
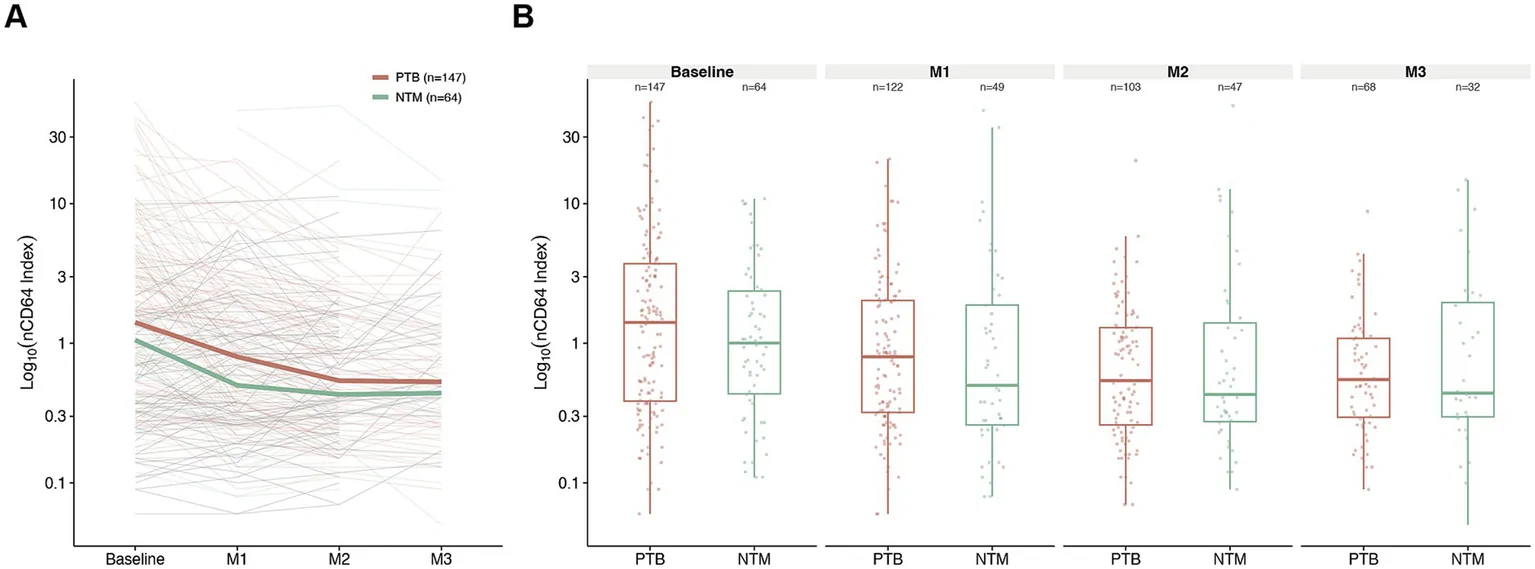
Longitudinal nCD64 index during anti-mycobacterial treatment in patients with PTB and NTM. **(A)** Individual trajectories across four time points (baseline, M1–M3) with median curves overlaid. **(B)** Box plots of nCD64 index at each time point for PTB and NTM. nCD64 index values are displayed on a log10 scale. PTB: all *p* < 0.001; NTM: all *p* < 0.05 within group comparisons. Paired sample sizes at each time point: PTB, M1: 122, M2: 103, M3: 68; NTM, M1: 49, M2: 47, M3: 32.

**Figure 7:**
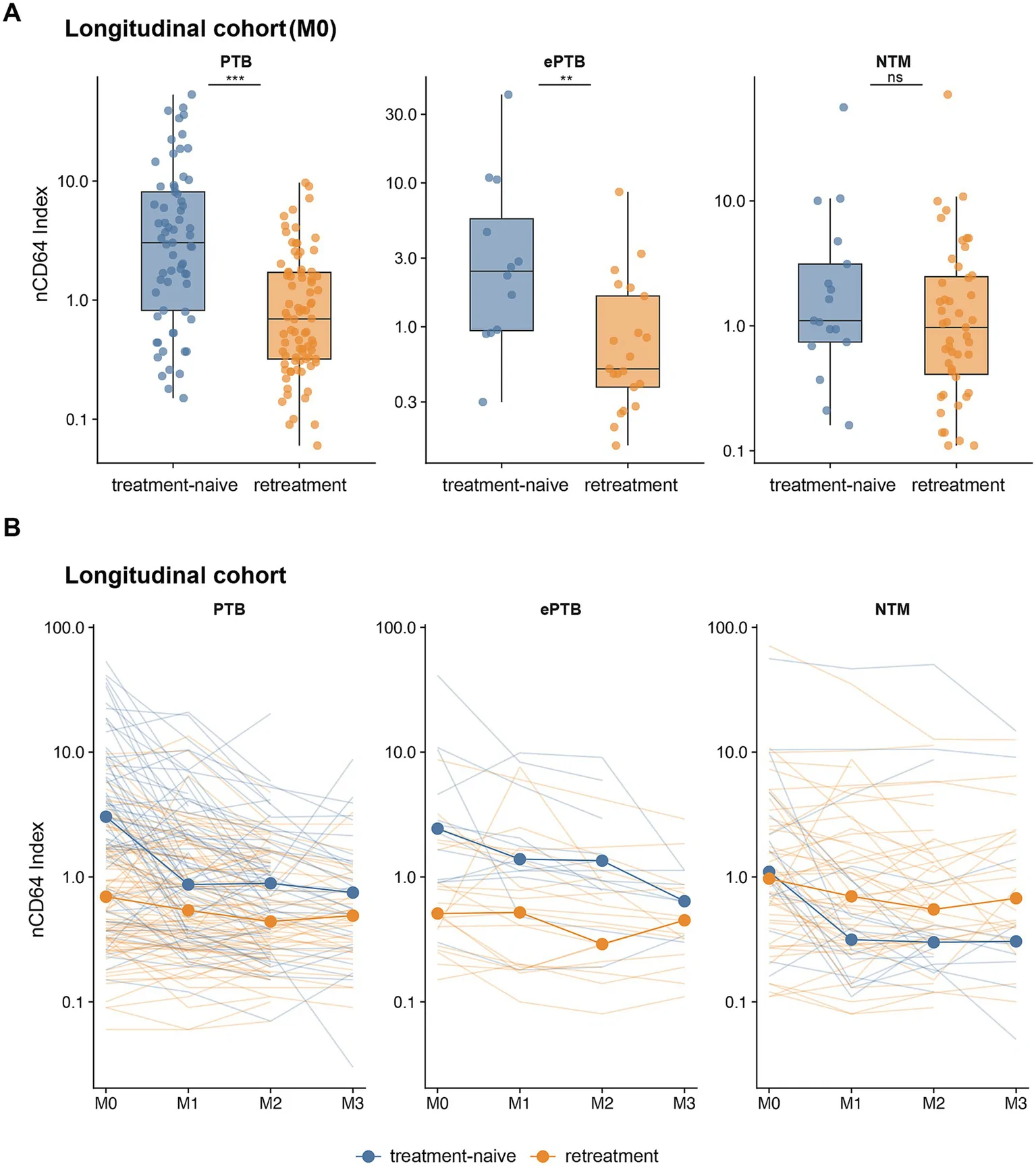
Effect of prior anti-mycobacterial treatment on baseline nCD64 index and longitudinal trajectory. **(A)** M0 nCD64 index in treatment-naive vs. retreatment patients. PTB (naive *n* = 65, retreatment *n* = 82), ePTB (naive *n* = 12, retreatment *n* = 21), and NTM (naive *n* = 17, retreatment *n* = 47). Pairwise comparisons by Mann–Whitney *U* test, **p < 0.01, ***p < 0.001. ns, not significant. **(B)** Longitudinal nCD64 index trajectories (M0–M3) stratified by treatment history for PTB, ePTB, and NTM. Bold lines indicate group medians; thin lines indicate individual trajectories. Linear mixed effects model tested Month × treatment history interaction, PTB: *p* < 0.001, ePTB: *p* = 0.079, NTM: *p* = 0.004. All values displayed on a log10 scale.

## Discussion

In this study, we evaluated nCD64 index in a treatment-naive cohort of five diagnostic groups and tracked its trajectory during antimicrobial therapy in an independent longitudinal cohort. First, nCD64 index discriminated ATB from both LTBI and NTM pulmonary disease (AUC = 0.739 and 0.744, respectively), the latter distinction not previously reported. Second, adding nCD64 index to routine inflammatory indicators improved discriminatory performance across all comparisons, indicating that it captures information not fully represented by WBC count or CRP. Third, nCD64 index declined significantly during treatment in both PTB and NTM patients, and treatment-naive patients exhibited steeper decline trajectories than retreatment patients. These results support the nCD64 index as a host-response marker that can complement microbiological and clinical assessment, with diagnostic and treatment-monitoring utility across the mycobacterial infection spectrum.

nCD64 index is rapidly upregulated by IFN-*γ* and G-CSF during active infection, preceding the rise of CRP and procalcitonin (PCT). This advantage has made nCD64 index an emerging candidate for early infection detection and treatment monitoring across diverse clinical settings, including autoimmune disease, lung infection, and pediatric critical illness (Li et al., 2009, 2026; Cao et al., 2022; Wu et al., 2025). *A prior* study demonstrated elevated nCD64 index in PTB and ePTB relative to pneumonia and healthy controls (Liu et al., 2020), and another study reported similar findings about nCD64 index in IGRA-positive individuals with active TB (Corrêa et al., 2019). However, the diagnostic value of nCD64 index across the spectrum of mycobacterial infections has not yet been fully characterized. Two clinical gaps are particularly relevant: one is that existing assays cannot distinguish between ATB and LTBI (Ni et al., 2026); the other is the overlap in clinical and radiological features between PTB and NTM, which makes differential diagnosis complicated.

The nCD64 index exhibits discriminatory capacity to differentiate active tuberculosis (ATB) from latent tuberculosis infection (LTBI), overcoming a major limitation of IGRA, which cannot reliably distinguish active from latent infection (Petruccioli et al., 2016). Prior work reported an AUC of 0.836 for the nCD64 index among IGRA-positive individuals (Corrêa et al., 2019), and multiple independent cohorts have demonstrated higher CD64 expression/transcription in ATB relative to LTBI (Berry et al., 2010; Nakiboneka et al., 2024). In our cohort, the nCD64 index yielded an AUC of 0.739 for discriminating ATB from LTBI. Notably, the nCD64 index and IGRA capture distinct components of the host immune response, and their diagnostic performance metrics cannot be directly cross-study compared. IGRA detects antigen-specific T-cell sensitization, which occurs in both ATB and LTBI, while the nCD64 index quantifies the inflammatory response triggered by ongoing mycobacterial replication. During active infection, IFN-*γ* and G-CSF rapidly upregulate surface CD64 expression on neutrophils. In LTBI, antigen-specific T-cell memory is established without persistent active bacillary replication, and systemic release of IFN-*γ* and G-CSF does not reach the threshold required for marked CD64 elevation (Petruccioli et al., 2016).

In the present cohort, ePTB patients exhibited a lower median nCD64 index than PTB patients, although the difference was not statistically significant (1.48 vs. 3.27, *p* = 0.518). This finding contrasts with previously reported markedly elevated nCD64 index values in ePTB patients (median 23.71) (Liu et al., 2020). This likely reflects compositional differences: their ePTB group included disseminated TB (18%), tuberculous pleuritis, and meningitis, whereas our ePTB cohort comprised localized manifestations (lymph node, skeletal, and urological TB) with lower systemic bacterial burden. However, our ePTB data exhibited a similar decline following anti-mycobacterial therapy (Supplementary Figures 1b,c).

PTB and NTM share substantial clinical and radiological overlap, making their differentiation a practical challenge. In our study, nCD64 index was significantly higher in PTB than NTM (*p* < 0.001), with an AUC of 0.744 for this comparison. Given that NTM infection elicits less pronounced neutrophil activation than PTB (Alkarni et al., 2023), the overall range of nCD64 index elevation is also narrower. The nCD64 index in PTB correlated moderately with common inflammation indicators whereas these associations were absent in NTM, suggesting that nCD64 index reflects a coordinated inflammatory response in PTB but a weaker and more attenuated pattern in NTM. TB-specific neutrophil CD64 upregulation has been directly demonstrated in PTB patients (Berry et al., 2010), supporting the interpretation that the higher nCD64 index in PTB reflects a more potent and specific neutrophil activation response to *M. tuberculosis*. Notably, nCD64 index levels did not differ significantly across NTM species subgroups (*M. intracellulare*, *M. abscessus*, *M. kansasii*, *M. avium* and rare species) in either the cross-sectional or longitudinal cohort (Supplementary Figure 3), indicating that the attenuated neutrophil response is a shared feature of NTM pulmonary disease rather than a species-specific phenomenon.

WBC count performed poorly across all diagnostic comparisons, consistent with its limited specificity. In our data, adding nCD64 index to WBC count improved AUC across all four comparisons: the largest gain was seen for ATB vs. LTBI and the improvement held after further adjustment for CRP. Together, these results indicate that nCD64 index may function as a practical complement to WBC count and CRP in the evaluation of mycobacterial infection. In the longitudinal cohort, CRP and ESR are not routinely monitored at scheduled follow-up visits for mycobacterial infections, and complete parallel trajectory data were therefore unavailable in this retrospective setting. Future prospective studies using multi-biomarker sampling protocols would allow more direct comparisons.

However, the nCD64 index showed limited ability to stratify clinical severity within each group: of 12 indicator-severity combinations examined, only one reached significance. A characteristic feature of tuberculosis-associated pleural effusion is a strong local IFN-*γ* response at the pleural interface, which continuously stimulates the upregulation of CD64 on systemic neutrophils, leading to elevated levels of nCD64 index in peripheral blood (Alemán et al., 2005; Mehraban et al., 2022).

The nCD64 index has previously been shown to reflect treatment response in bacterial infections (Dimoula et al., 2014), and reductions in the nCD64 index as early as week 1 of anti-mycobacterial therapy have been documented (Liu et al., 2020). Our longitudinal analysis further confirmed this observation, and extended comparable observations to the NTM group in which longitudinal cohort data are scarce. A previous study of 9 patients reported that nCD64 index discriminated active from inactive NTM disease, but treatment response tracking has not been performed (Komiya et al., 2014). Although treatment regimens differed, the two groups exhibited a similar descending trend, suggesting that nCD64 index tracks resolution of neutrophil activation broadly rather than any pathogen-specific process. The ePTB group showed a similar trend, though the smaller sample size limits firm conclusions.

Compared with treatment-naive patients, patients with retreatment PTB and ePTB had significantly lower baseline nCD64 index; linear mixed-effects modelling revealed a significant Month × treatment history interaction in PTB (*p* < 0.001) and NTM (*p* = 0.004). This pattern is consistent with impaired IFN-*γ*-driven neutrophil priming following prolonged antigen exposure, which may reflect T-cell exhaustion (Jayaraman et al., 2016; Lombardi et al., 2021). These findings suggest that nCD64 index may be particularly informative for monitoring initial treatment response in treatment-naive patients, where the range of decline is most pronounced.

While traditional diagnostic methods through culture, species identification, and drug susceptibility testing remain essential for conclusive diagnosis and treatment regimens, the nCD64 index may serve as an early complementary indicator to support clinical triage before microbiological results are available. However, our study had some limitations. First, the ePTB subgroup was relatively small, and IGRA was not performed in the HD group. Also, a small proportion of individuals with undiagnosed LTBI may have been included, which would result in a conservative underestimation of diagnostic performance. Second, the single-center design at a specialized referral hospital may limit the generalizability of our findings. In addition, categorical clinical severity features have inherently limited sensitivity for capturing disease heterogeneity. Furthermore, residual confounding including diabetes, cannot be entirely excluded in this retrospective study. Demographic characteristics including age and sex differed across the groups, partly reflecting differences in the source populations. A contribution of age or sex to group-level variation in nCD64 index cannot be entirely excluded. The diagnostic cutoffs identified by ROC analysis were derived from and applied to the same cohort without external validation. Prospective validation in larger, multi-center cohorts with more comprehensive clinical features including drug resistance status is warranted.

## Conclusion

Our two-cohort study demonstrated that nCD64 index discriminates ATB from LTBI and NTM, adds value beyond routine inflammatory markers, and tracks serial changes during anti-mycobacterial treatment across the mycobacterial infection spectrum. Notably, treatment-naive patients exhibited steeper decline trajectories than retreatment patients, supporting the utility of nCD64 index for tracking initial treatment response. As a rapid, non-invasive, and standardizable method, the nCD64 index warrants evaluation as a complementary tool to support early clinical triage. Nevertheless, traditional diagnostic methods, particularly microbiological confirmation and species identification, remain indispensable for conclusive diagnosis and treatment decisions.

## Data Availability

The raw data supporting the conclusions of this article will be made available by the authors, without undue reservation.
