# Supplementary material for: Neutrophil CD64 index for diagnosing and tracking treatment response across the mycobacterial infection spectrum: a retrospective two-cohort study
Source: Front Microbiol. 2026 Sep 25;17:1980277. doi: 10.3389/fmicb.2026.1980277 (PMC13649758; doi:10.3389/fmicb.2026.1980277)
Supplement: Supplementary file 1 [file Data_Sheet_1.PDF]

Supplementary Material

Supplementary Figures

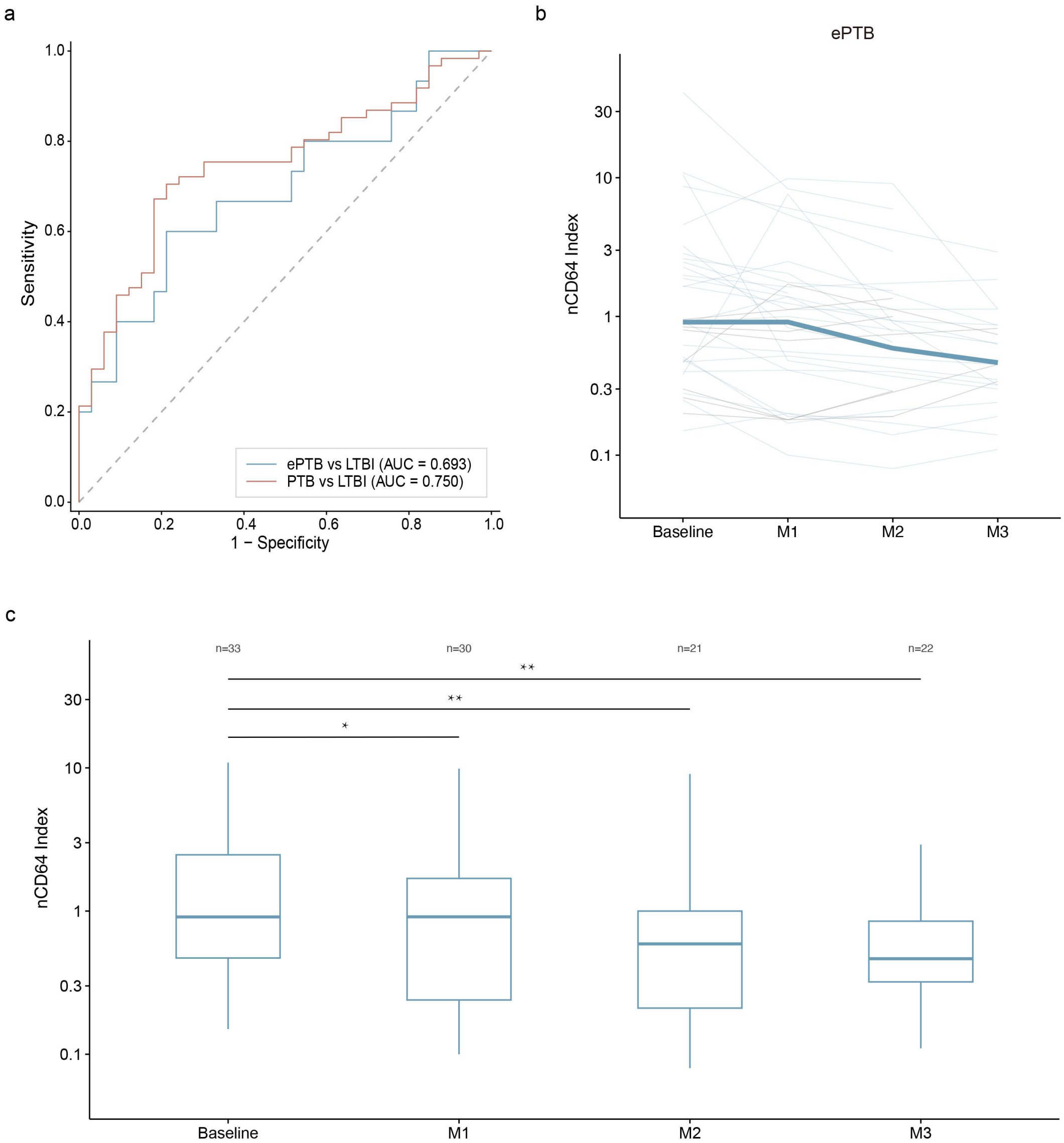

Supplementary Figure 1. Analyses for the ePTB subgroup. (a) ROC analysis of nCD64 index for ePTB diagnosis. ROC curves comparing ePTB (n=15) against LTBI (n=33), with PTB vs LTBI shown as reference. AUC, area under the ROC curve; ROC, receiver operating characteristic. (b) Individual trajectories of nCD64 index during anti-microbial treatment in ePTB patients (n=33), with group median overlaid; decreasing trajectories in blue, increasing trajectories in grey. (c) Box plots of nCD64 index at M0 (baseline), M1, M2, and M3. Paired sample sizes at each time point: M1: 30, M2: 21, M3: 22.

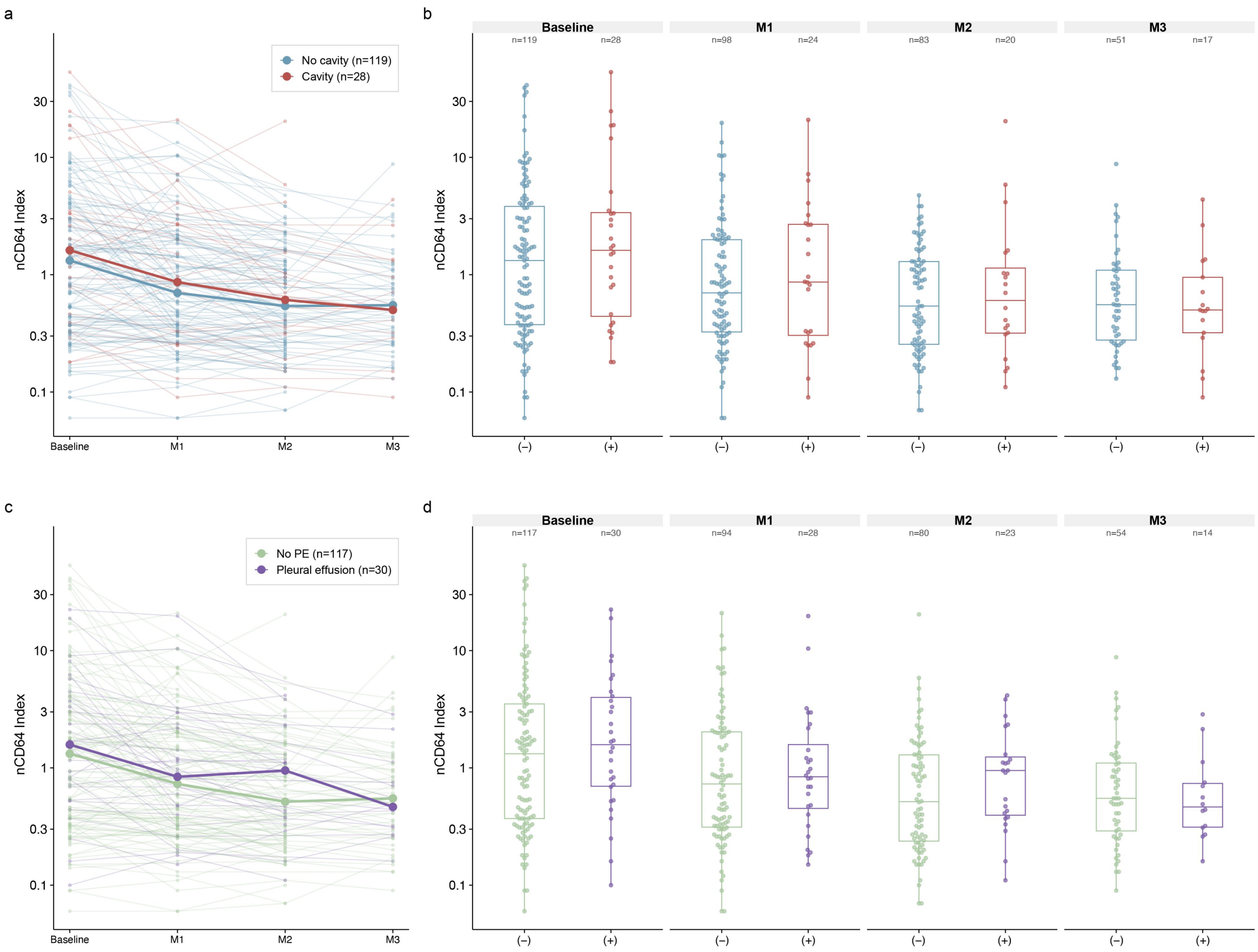

Supplementary Figure 2. Longitudinal nCD64 index in PTB patients stratified by baseline clinical features. (a) Individual trajectories and (b) box plots at M0–M3 for PTB patients with cavity (+) or without cavity (–). (c) Individual trajectories and (d) box plots at M0–M3 for PTB patients with PE (+) or without PE (–). Between-group comparisons at each time point by Mann-Whitney U test; LME (linear mixed-effects) model tested Month × clinical feature interaction. ns: not significant. PE: pleural effusion.

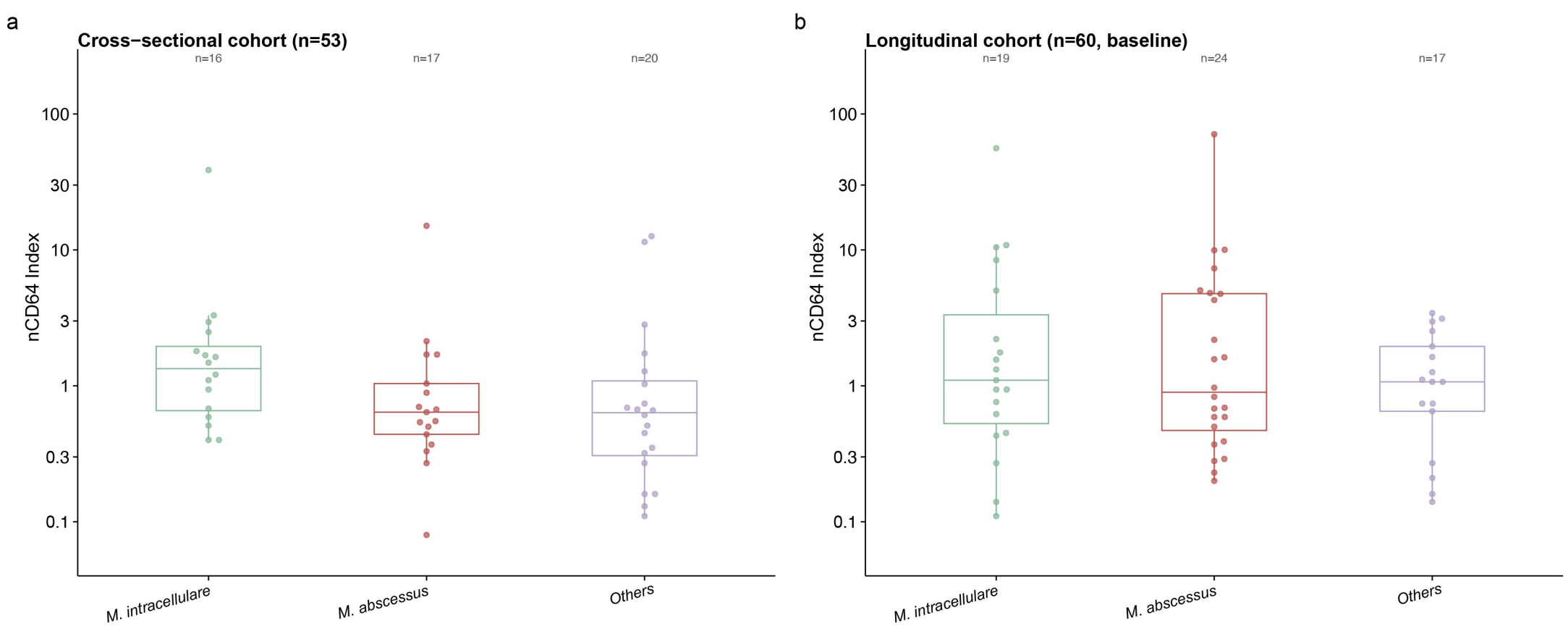

Supplementary Figure 3. nCD64 index across NTM species. (a) Cross-sectional cohort (n=53). (b) Longitudinal cohort at M0 baseline (n=60). Sample sizes for each

species are annotated above the respective box. NTM patients were categorized by identified mycobacterial species into M. intracellulare, M. abscessus, and others (including M. kansasii, M. avium, and rare species); patients with unrecorded or unknown strain information were excluded from the plot but were retained in all primary analyses. Comparisons between species were performed by Kruskal-Wallis test, no significant differences were observed (all p>0.05).

Supplementary tables

Supplementary Table 1 Comparion of performance of nCD64 index across the different diagnostic groups

Comparison	n (case/ctrl)	AUC (95% CI)	Cutoff	Sensitivity (%)	Specificity (%)	PLR	NLR
ATB vs HD	76/40	0.805 (0.726–0.883)	0.98	73.7%	85.0%	4.91	0.31
ATB vs LTBI	76/33	0.739 (0.643–0.834)	1.31	68.4%	78.8%	3.23	0.4
ATB vs NTM	76/55	0.733 (0.646–0.819)	1.84	61.8%	83.6%	3.77	0.46
ATB vs PI	76/44	0.742 (0.652–0.831)	2.09	59.2%	81.8%	3.25	0.5
PTB vs NTM	61/55	0.744 (0.652–0.837)	1.84	65.6%	83.6%	4.0	0.41
PTB vs PI	61/44	0.752 (0.660–0.845)	2.09	62.3%	81.8%	3.42	0.46
ePTB vs LTBI	15/33	0.693 (0.520–0.866)	1.31	60.0%	78.8%	2.83	0.51
PTB vs LTBI	61/33	0.750 (0.650–0.850)	1.34	70.5%	78.8%	3.33	0.37

Abbreviations: ATB, active tuberculosis; PTB, pulmonary tuberculosis; ePTB, extrapulmonary tuberculosis; LTBI, latent tuberculosis infection; NTM, non-tuberculous mycobacteria; PI, bacterial pulmonary infection; HD, healthy donors; AUC, area under the ROC curve; PLR, positive likelihood ratio; NLR, negative likelihood ratio.

Supplementary Table 2 Incremental diagnostic value of the nCD64 index in each comparison group

baseline model	Comparison	n (case/ctrl)	Enhanced model	Cutoff	Sensitivity (%)	Specificity (%)	PLR	NLR	*P* value
WBC (full cohort)	PTB vs NTM	61/55	WBC + nCD64 index	0.50	77%	76.4%	3.26	0.3	0.014
WBC (full cohort)	ATB vs LTBI	76/33	WBC + nCD64 index	0.66	72.4%	75.8%	2.99	0.36	0.004

WBC (full cohort)	PTB vs PI	61/44	WBC + nCD64 index	0.62	63.9%	81.8%	3.51	0.44	<0.001
WBC (full cohort)	ATB vs NTM	76/55	WBC + nCD64 index	0.57	69.7%	78.2%	3.2	0.39	0.006
WBC + CRP (CRP subset)	PTB vs NTM	55/32	WBC + CRP + nCD64 index	0.63	70.9%	81.2%	3.77	0.36	0.106
WBC + CRP (CRP subset)	ATB vs LTBI	68/25	WBC + CRP + nCD64 index	0.68	75%	76%	3.12	0.33	0.08
WBC + CRP (CRP subset)	PTB vs PI	55/29	WBC + CRP + nCD64 index	0.55	81.8%	62.1%	2.16	0.29	0.003
WBC + CRP (CRP subset)	ATB vs NTM	68/32	WBC + CRP + nCD64 index	0.72	60.3%	87.5%	4.82	0.45	0.082

Abbreviations: ATB, active tuberculosis; PTB, pulmonary tuberculosis; LTBI, latent tuberculosis infection; NTM, non-tuberculous mycobacteria; PI, bacterial pulmonary infection; WBC, white blood cell; CRP, C-reactive protein; PLR, positive likelihood ratio; NLR, negative likelihood ratio.
